# Bimanual Microincision Cataract Surgery versus Coaxial Microincision Cataract Surgery: A Meta-Analysis of Randomized Controlled Trials and Cohort Studies

**DOI:** 10.1155/2017/3737603

**Published:** 2017-08-23

**Authors:** Chenxi Fu, Naipin Chu, Xiaoning Yu, Ke Yao

**Affiliations:** Eye Center, Second Affiliated Hospital, School of Medicine, Zhejiang University, Hangzhou, China

## Abstract

**Purpose:**

This meta-analysis was conducted to compare the intraoperative and postoperative outcomes of bimanual microincision cataract surgery (B-MICS) and coaxial microincision cataract surgery (C-MICS).

**Methods:**

Three databases were searched for papers that compared B-MICS and C-MICS from inception to June 2016. The following intraoperative and postoperative outcomes were included in the final meta-analysis: ultrasound time (UST), effective phacoemulsification time (EPT), balanced salt solution use (BSS use), mean surgery time, best-corrected visual acuity (BCVA), central corneal thickness (CCT), and increased CCT.

**Results:**

There were no statistically significant differences in mean surgery time, UST, BSS use, BCVA, CCT, or increased CCT (one subgroup at postoperative day 7-8 and another subgroup at postoperative day 30). However, there was less EPT needed during surgery (*p* < 0.01) and lower levels of increased CCT at postoperative day 1 (*p* = 0.02) in the B-MICS group compared with the C-MICS group.

**Conclusions:**

The EPT was shorter and increased CCT was less at postoperative day 1 in the B-MICS group. There were no statistically significant differences in other intraoperative and postoperative outcomes between the B-MICS group and the C-MICS group. B-MICS is an efficient and safe cataract surgery procedure.

## 1. Introduction

With the development of equipment for cataract surgery and increased requirements for visual outcome, the main recent change in cataract surgical procedure aims to decrease the size of the clean corneal incision. In coaxial microincision cataract surgery (C-MICS), irrigation, aspiration, and phacoemulsification are performed with the same instruments, which is similar to standard coaxial small incision cataract surgery (C-SICS) but provides a smaller incision [1, 2]. Less surgically induced astigmatism (SIA) and faster wound healing are expected from incision sizes below 2.2 mm, which makes C-MICS more popular among ophthalmologists around the world [3]. For B-MICS, the separate irrigating hand piece port can be supplementary during phacoemulsification, and the same size of two incisions and hand piece ports makes the interchange of the two ports possible during surgery [4, 5]. However, the extra step of enlarging the main incision or making a third incision is a drawback of the pervasiveness of the B-MICS.

According to a recent study [6], there are new aspheric intraocular lenses that are small enough to fit through a 1.4 mm incision, which saves the trouble of having an extra step for the IOL implant. Since the trend in cataract surgery has been to minimize the corneal incision, the 1.4 mm incision of B-MICS may have advantages in refractive surgery [4, 5]. Two published meta-analysis studies have compared the outcomes of B-MICS versus C-SICS and C-MICS versus C-SICS. To our knowledge, there has not been a meta-analysis comparing the outcomes of B-MICS and C-MICS [7, 8]. Several clinical studies have compared the intraoperative and postoperative outcomes of B-MICS and C-MICS, but there has been no clear conclusion. This meta-analysis was performed to compare the outcomes of B-MICS with C-MICS to make recommendations for improvements in cataract surgery.

## 2. Materials and Methods

This meta-analysis was performed according to the Preferred Reporting Items for Systematic Reviews and Meta-Analyses (PRISMA) statement [9].

The following terms were searched in PubMed, Web of Science, and the Cochrane Library: “bimanual microincision,” “biaxial microincision,” “bimanual microincisional cataract surgery,” “bimanual phacoemulsification,” “biaxial phacoemulsification,” “coaxial microincision,” “coaxial microincisional cataract surgery,” and “coaxial phacoemulsification.” Papers published before July 2016 were included in the search. The references of the included papers were reviewed to seek papers that were missed in the primary search. The papers included in the meta-analysis met the following criteria: (1) the incision of the coaxial microincision cataract surgery (C-MICS) was less than 2.2 mm and (2) patients in the study had no other ocular diseases other than cataracts. Two investigators (Fu and Chu) searched the three databases in all fields independently.

**Figure 1:**
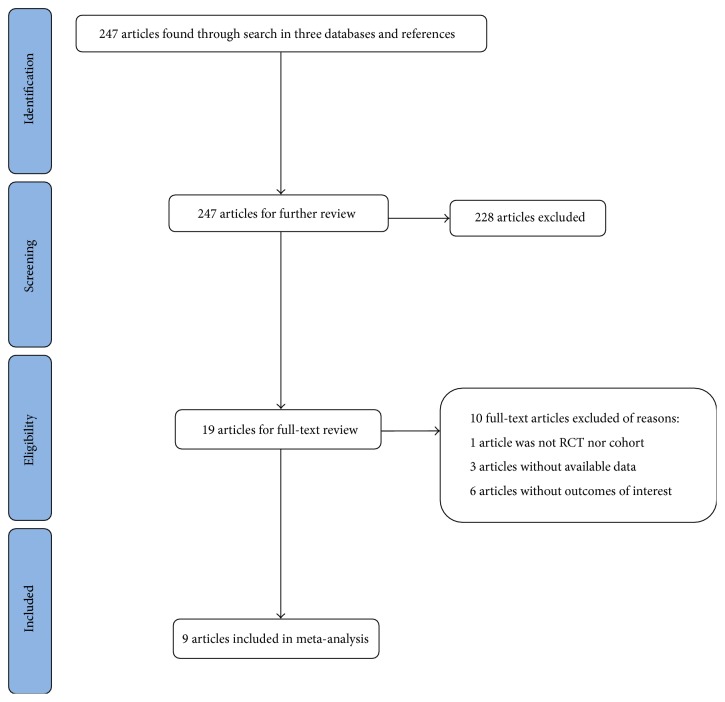
Flow diagram of study selection progress.

After scanning the titles and abstracts, Fu and Chu obtained access to the full text of the papers that compared outcomes of C-MICS and B-MICS. After reading the full text, papers were included in the meta-analysis based on the criteria listed above. The search and exclusion process was conducted as shown in Figure 1. The basic information and the data on intraoperative and postoperative outcomes, including EPT, mean surgery time, BBS use, UST, BCVA, CCT, and increased CCT, were extracted from the papers independently by two investigators. If there were any disagreements, Fu and Chu double-checked the related papers for data verification. For B-MICS, the sizes of the first-made incision and the extended or the third incision for the IOL implant were both recorded if they were reported in the papers. The BCVA data extracted from all papers were reported in the logMAR system. To avoid extracting repetitious data, two investigators double-checked the sources and characteristics of the patients in the studies with the same subject and author. Seven RCTs were evaluated according to the Jadad score system, and studies of three or more points were of good quality.

The data on all outcomes were analysed using Stata software (version 12.0, StataCorp, College Station, TX, USA). The means and SDs of outcomes were extracted from the papers to obtain the weighted mean difference (WMD) with a 95% confidence interval (CI). Except for heterogeneity and metaregression analyses of the data, the statistical significance level was set at *p* < 0.01. For heterogeneity among studies, the significance level was set at *p* < 0.10 for Cochran's *Q* statistic and *I*^2^ > 50% for the *I*^2^ index score [10]. A fixed-effects model based on the inverse variance method was used for continuous data unless the heterogeneity among the studies was high (I^2^ score > 50%), in which case, a random-effects model based on the DerSimonian and Laird method was used [11]. To assess the robustness of the results, a sensitivity analysis was performed on all results by excluding one study at a time. Egger's linear regression and Begg's rank correlation tests were performed to assess the potential publication bias [12, 13]. The statistical analysis procedures described above were repeated by two investigators independently.

## 3. Results

Among the included papers, there were seven randomized controlled trials (RCTs) [3, 14–19] and two cohort studies [20, 21]. Basic information on the nine articles is provided in Table 1. The included studies reported on a total of 711 eyes (356 eyes in the B-MICS group and 355 eyes in the C-MICS group).

### 3.1. Intraoperative Outcomes

#### 3.1.1. Effective Phacoemulsification Time (EPT)

**Figure 2:**
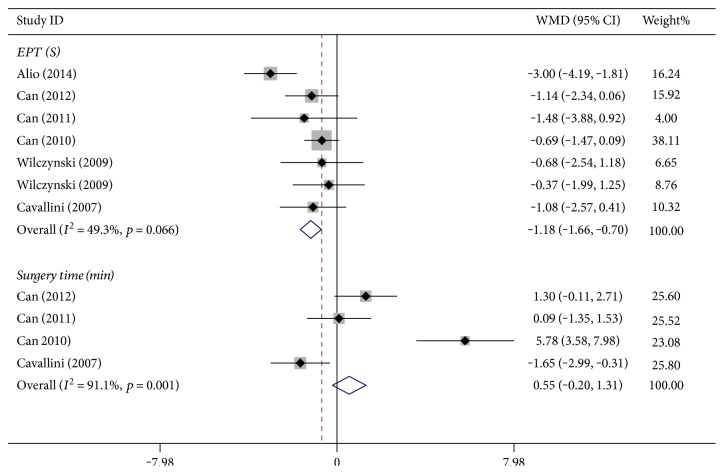
Effective phacoemulsification time and surgery time between bimanual microincision cataract surgery and coaxial microincision cataract surgery.

In seven studies reporting on 589 eyes, the EPT was longer in the C-MICS group than in the B-MICS group in the fixed-effects model (Figure 2, WMD: −1.18, 95% CI: −1.66 to −0.70, *p* ≤ 0.001, *I*^2^ = 49.3%, *P*_heterogeneity_ = 0.066).

#### 3.1.2. Mean Surgery Time

In four studies that reported on 330 eyes, the mean surgery time was recorded. The forest plot showed that there was no statistically significant difference between the B-MICS group and the C-MICS group in the random-effects model (Figure 2, WMD: 0.55, 95% CI: −0.20 to 1.31, *p* = 0.338, *I *^2^ = 91.1%, *P*_heterogeneity_ = 0.001).

#### 3.1.3. Use of Balanced Salt Solution (BSS)

**Figure 3:**
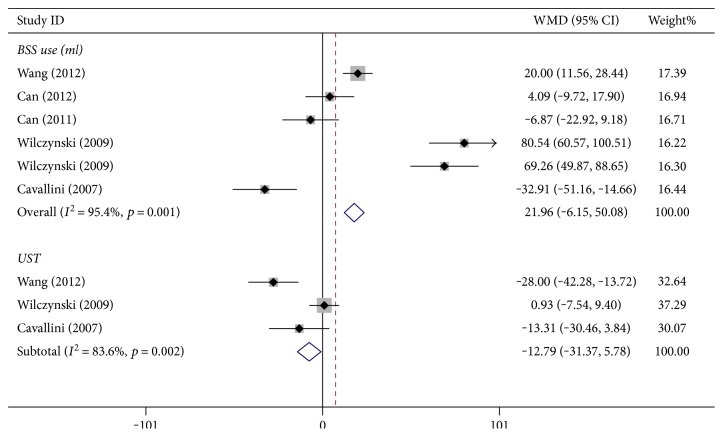
Balanced saline use and ultrasound time between bimanual microincision cataract surgery and coaxial microincision cataract surgery.

As shown in Figure 3, there was no statistically significant difference in BBS use in the random-effects model (Figure 3, WMD: 21.963, 95% CI: −6.150 to 50.076, *p* = 0.126, *I*^2^ = 95.4%, *P*_heterogeneity_ = 0.001).

#### 3.1.4. Ultrasound Time (UST)

Three studies reported UST, and significant heterogeneity in UST among these studies was found. As shown in Figure 3, there was no statistically significant difference between the two groups in the random-effects model (Figure 3, WMD: −12.79, 95% CI: −31.37 to 5.78, *p* = 0.177, *I*^2^ = 83.6%, *P*_heterogeneity_ = 0.002).

### 3.2. Postoperative Outcomes

#### 3.2.1. Best-Corrected Visual Acuity (BCVA)

**Figure 4:**
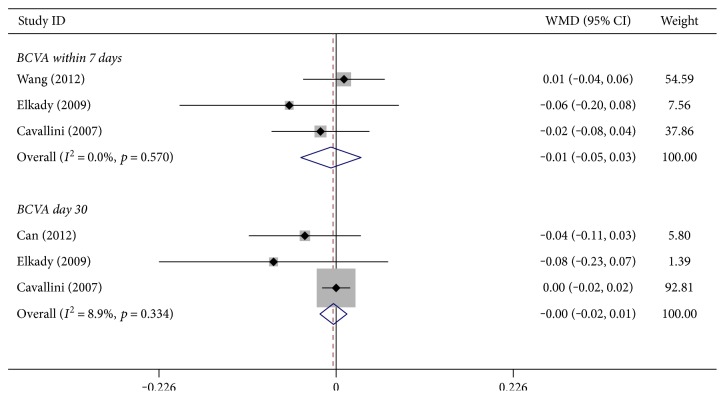
Best-corrected visual acuity between bimanual microincision cataract surgery and coaxial microincision cataract surgery.

In five studies, the BCVA was measured at postoperative day 1, day 7, day 30, day 60, and day 90. Subgroup meta-analysis was performed for two subgroups: BCVA within 7 postoperative days (222 eyes) and BCVA at day 30 (160 eyes). In both groups, no statistically significant difference was found between the B-MICS group and the C-MICS group in the fixed-effects model (Figure 4, within 7 days: WMD: −0.007, 95% CI: −0.045 to 0.032, *p* = 0.735, *I*^2^ = 0.0%, *P*_heterogeneity_ = 0.570; at day 30: WMD: −0.003, 95% CI: −0.021 to 0.014, *p* = 0.697, *I*^2^ = 8.9%, *P*_heterogeneity_ = 0.334).

#### 3.2.2. Central Corneal Thickness

**Figure 5:**
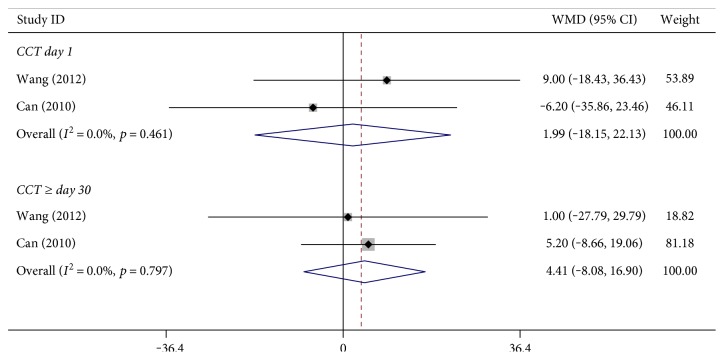
Increased central corneal thickness between bimanual microincision cataract surgery and coaxial microincision cataract surgery.

CCT was measured in three studies. We used data from two studies that recorded the precise CCT measuring time for 82 and 90 eyes, respectively, and analysed the postoperative CCT in the day 1 subgroup and the after day 30 subgroup. As shown in the forest plot, no statistically significant difference was found in either subgroup (Figure 5, at day 1: WMD: 1.991, 95% CI: −18.148 to 22.130, *p* = 0.846, *I*^2^ = 0.0%, *P*_heterogeneity_ = 0.461; after day 30: WMD: 4.409, 95% CI: −8.081 to 16.899, *p* = 0.479, *I*^2^ = 0.0%, *P*_heterogeneity_ = 0.797).

#### 3.2.3. Increased Central Corneal Thickness

**Figure 6:**
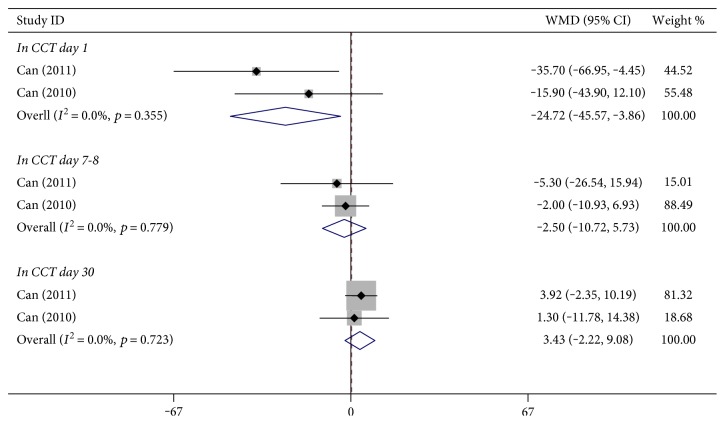
Central corneal thickness between bimanual microincision cataract surgery and coaxial microincision cataract surgery.

In two studies, one reporting on 60 eyes and one reporting on 90 eyes, increased CCT was measured and calculated. Three subgroups (day 1, day 7-8, and day 30) were assessed. There were no statistically significant differences in the day 7-8 subgroup or in the day 30 subgroup. And for the subgroup day 1, the forest plot showed that increased CCT was less common in the B-MICS group than in the C-MICS group in the fixed-effects model (Figure 6, day 1: WMD: −24.715, 95% CI: −45.569 to −3.861, *p* = 0.020, *I*^2^ = 0.0%, *P*_heterogeneity_ = 0.355; day 7-8: WMD: −2.495, 95% CI: −10.724 to 5.733, *p* = 0.552, *I*^2^ = 0.0%, *P*_heterogeneity_ = 0.779; day 30: WMD: 3.431, 95% CI: −2.223 to 9.085, *p* = 0.903, *I*^2^ = 0.0%, *P*_heterogeneity_ = 0.903).

### 3.3. Sensitivity Analysis and Publication Bias

After excluding one study at a time, the results of different outcomes all fell in the 95% CI of all articles, except for the postoperative BCVA at day 30 (estimate −0.477, 95% CI: −0.2067 to 0.0138).

No publication biases were found for any of the results of the intraoperative and postoperative outcomes.

## 4. Discussion

For the pooled results of the B-MICS and C-MICS in this meta-analysis, no statistically significant differences were found in mean surgery time, UST, BSS use, postoperative BCVA (within 7 days and at day 30), postoperative CCT (at day 1 and after day 30), and postoperative increased CCT (at day 7-8 and day 30). Figure 2 demonstrates that less EPT was needed in the B-MICS group. Less increased CCT at postoperative day 1 was found in the B-MICS group, as shown in Figure 6.

The shorter EPT in B-MICS may be due to the “cold” phacoemulsification mode used in B-MICS, or it may be due to the separation of the irrigation port from the aspiration port in B-MICS, which can avoid a competing current from the phacoemulsification tip and assist in the emulsification and aspiration process. Moreover, the separation and exchangeable hand pieces can provide surgeons with more flexibility to clean the subincisional cortex and residual viscoelastic material [4, 5, 19, 22]. The decrease in EPT caused less damage to the cornea, which was reflected by lower levels of postoperative increased CCT. The shorter EPT and the lower levels of postoperative increased CCT in the B-MICS group may accelerate the healing of the corneal wound and reduce the endothelial cell loss percentage (ECL %).

The intraoperative and postoperative complications were not analysed because the incidence of intraoperative complications was quite low, and long-term postoperative complications such as posterior capsule opacification (PCO) were rarely found during follow-up in the included studies. PCO develops several months to a few years after uneventful cataract surgeries, but the longest follow-up time in the included studies was three months. Among all cases reported in the studies, three cases in the B-MICS group (6.6%) and one (2.2%) in the C-MICS group from the Can et al. study suffered from intraoperative complications [16]. In all four cases, IOL were implanted successfully during the surgery, and related postoperative complications were not reported. Can et al. also reported postoperative complications in three cases (anterior chamber inflammation in 2 eyes, posterior capsule opacification in 1 eye). No intraoperative or postoperative complications were reported in the other studies. It seems that there were more complications in the B-MICS group. However, in some studies [23–25], less posterior wound retraction, less intraoperative and postoperative inflammation, and lower risk of endophthalmitis were observed, which implied a faster and better recovery of the corneal wound. Based on the sample size and number of studies included, studies with large sample sizes and with longer follow-up times are necessary for the comparison of the two surgery techniques.

Four papers included in this meta-analysis reported SIA [14, 16, 18], which could not be analysed statistically because the SIA measurement time points were different and the studies adopted two different methods for IOL implant. Lower levels of SIA were found in the B-MICS group in the Cavallini et al. study [14] (at postoperative day 7 and 1 month), the Can et al. study [16] (at postoperative 3 months), and the Can et al. study [18] (at postoperative 1 month), whereas higher levels of SIA were reported in the Cavallini et al. study [14] (at postoperative day 1 and 3 months) and the Wilczynski et al. study [21] (at postoperative day 1). However, no difference was observed in postoperative clinical visual quality. In some earlier studies [15, 26], there were no statistically significant differences found in corneal power and postoperative astigmatism changes between the B-MICS and C-MICS group; the authors also claimed that both techniques were able to provide an astigmatically neutral incision. The IOL implant procedure of B-MICS in the included studies all required the step of expansion of the initial incision or making a third incision. Because the smaller corneal wound is known to be associated with lower levels of SIA [27, 28], the use of the new IOL injectable directly through a 1.4 mm incision, which allows surgeons to skip the extra damage step to the cornea, may cause less SIA than C-MICS with a 1.8 mm corneal incision.

Because the incision sizes of B-MICS and C-MICS are both micro, SIA or BCVA may not be precise enough to detect differences between the two procedures. In three studies [15, 17, 18], corneal optical coherence tomography and corneal topography were used to measure parameters such as the detachment rate of the Descemet's membrane, endothelial gaps, epithelial gaps, and surgery-induced corneal coma to compare the two microincision techniques. Lower levels of surgery-induced corneal coma were reported in the B-MICS group in one study [17], which was consistent with the results of the Eliwa and Hamza study [29].

Although the incision of cornea is 2.0 mm or less in the original definition of MICS [30, 31], a few papers extended the range to 2.2 mm due to difference in machine settings and the following phacoemulsification and implantation procedures enlarging the incision a little bit. So, we adopted less than 2.2 mm as the range of MICS in this paper. Among all results, high heterogeneity was detected in mean surgery time, UST, and BSS use. The high levels of heterogeneity may be due to the number of papers reporting data on individual outcomes, characteristics of patients, and locations of different studies. Our study was limited by the number of studies available and the data recorded in each study. Only a few studies included analysis of the outcomes of mean surgery time, EPT, UST, CCT, and increased CCT. Further studies are needed to confirm the conclusions of our meta-analysis. We were not able to conduct statistical analyses for some postoperative outcomes, such as SIA, average ultrasound power (AVE), and ECL%. Further studies comparing the two techniques are necessary.

There are some advantages to our study. All nine studies included in this meta-analysis were prospective, and seven of the studies were randomized trials. The operations in the B-MICS and C-MICS groups were performed by the same experienced surgeon in the majority of the included studies [3, 14–18], which avoided the bias of the proficiency level of different surgeons. For the data on postoperative outcomes such as BCVA, CCT, and increased CCT, subgroup analyses were performed on the basis of time slot of follow-up, which avoided the bias of different follow-up times.

The learning curve is also considered to be a drawback of the pervasiveness of B-MICS. Some related studies have been conducted. In one long-term follow-up study comparing the clinical results, such as PCO incidence and clear corneal incision (CCI) architecture of B-MICS, between surgeons in training and experienced surgeons [32], the PCO incidence was higher and corneal incisions were shorter and less angled for less experienced surgeons. The higher PCO incidence may be due to insufficient cleaning of the posterior capsule; the different CCI architectures may be induced by the difficulty in using the nondominant hand. However, in the long-term follow-up, there were no statistically significant differences in BCVA, SIA, and corneal pachymetry changes between surgeons in training and experienced surgeons. This study indicated that there was a process of experience accumulation for inexperienced surgeons. However, there was no such difference in skill acquisition between the two techniques. One recent study reported no significant difference in visual outcomes and complication rates between B-MICS performed by surgeons in training and C-MICS performed by surgeons in training that reported before [33]. Therefore, the results of both studies suggest that B-MICS can be considered as a safe and effective surgery performed by surgeons in training.

In this meta-analysis, there was a shorter EPT and lower levels of increased CCT at postoperative day 1 in the B-MICS group, but other main clinical outcomes of B-MICS and C-MICS were not significantly different. Thus, both cataract surgery techniques are efficient, safe, and appropriate for cataract surgery.

## Figures and Tables

**Table 1:** Characteristics of 9 studies included in the meta-analysis.

Source (publication year, country)	Number of eyes	Age (year)	Gender (M/F)	First incision size	Final incision size	Follow-up (day)	Jadad score
	B-MICS/C-MICS	B-MICS/C-MICS	B-MICS/C-MICS	B-MICS/C-MICS	B-MICS/C-MICS		
Cavallini et al. (2007, Italy)	50/50	NA	Total: 15/35	1.4/2.2	2.24 ± 0.04/2.29 ± 0.08	90	1 + 1-0 + 0 + 0-1 + 1
Wilczynski et al. (2009, Poland)	50/58	67.8 ± 9.5/73.8 ± 8.4	(35/15)/(33/25)	1.7/1.8	NA	30	
Wilczynski et al. (2009, Poland)	50/51	67 ± 10 /73 ± 8	(9/41)/(19/32)	1.7/1.8	NA	30	
Elkady et al. (2009, Spain)	25/15	1.73 ± 0.08/2.24	(5/11)/(9/9)	1.4/2.2	1.73 ± 0.08/2.24	30	1 + 1-0 + 0 + 0-1 + 1
Can et al. (2010, Turkey)	45/45	61.5 ± 8.1/65.8 ± 13.2	(17/14)/(14/18)	NA	1.89 ± 0.21/2.26 ± 0.07	90	1 + 0-1 + 0 + 0-0 + 1
Can et al. (2011, Turkey)	30/30	63.6 ± 15.5/69.1 ± 9.1	(13/12)/(13/13)	1.2–1.4/1.6–1.8	NA	30	1 + 1-0 + 0 + 0-1 + 1
Can et al. (2012, Turkey)	40/40	65.29 ± 8.24/63.59 ± 11.77	(16/12)/(17/15)	1.2–1.4/1.6–1.8	NA	30	1 + 1-0 + 0 + 0-1 + 1
Wang et al. (2012, China)	41/41	Total: 67 ± 10	NA	1.3/2.2	NA	30	1 + 0-1 + 0 + 0-0 + 1
Alió et al. (2014, Egypt)	25/25	67.60 ± 8.46/70.50 ± 8.88	NA	1.0/2.2	NA	30	1 + 1-0 + 0 + 0-1 + 1

## References

[1] Osher R. H., Injev V. P. (2007). Microcoaxial phacoemulsification part 1: laboratory studies. *Journal of Cataract and Refractive Surgery*.

[2] Alio J. L., Rodriguez-Prats J. L., Vianello A., Galal A. (2005). Visual outcome of microincision cataract surgery with implantation of an Acri. Smart lens. *Journal of Cataract and Refractive Surgery*.

[3] Wang Y., Xia Y., Liu X., Zheng D., Luo L., Liu Y. (2012). Comparison of bimanual and micro-coaxial phacoemulsification with torsional ultrasound. *Acta Ophthalmologica*.

[4] Paul T., Braga-Mele R. (2005). Bimanual microincisional phacoemulsification: the future of cataract surgery?. *Current Opinion in Ophthalmology*.

[5] Weikert M. P. (2006). Update on bimanual microincisional cataract surgery. *Current Opinion in Ophthalmology*.

[6] von Sonnleithner C., Bergholz R., Gonnermann J., Klamann M. K., Torun N., Bertelmann E. (2015). Clinical results and higher-order aberrations after 1.4-mm biaxial cataract surgery and implantation of a new aspheric intraocular lens. *Ophthalmic Research*.

[7] Chen C., Zhu M., Sun Y., Qu X., Xu X. (2015). Bimanual microincision versus standard coaxial small-incision cataract surgery: meta-analysis of randomized controlled trials. *European Journal of Ophthalmology*.

[8] Shentu X., Zhang X., Tang X., Yu X. (2016). Coaxial microincision cataract surgery versus standard coaxial small-incision cataract surgery: a meta-analysis of randomized controlled trials. *PLoS One*.

[9] Moher D., Liberati A., Tetzlaff J., Altman D. G., PRISMA Group (2010). Preferred reporting items for systematic reviews and meta-analyses: the PRISMA statement. *International Journal of Surgery*.

[10] Higgins J. P., Thompson S. G., Deeks J. J., Altman D. G. (2003). Measuring inconsistency in meta-analyses. *British Medical Journal*.

[11] Der Simonian R., Kacker R. (2007). Random-effects model for meta-analysis of clinical trials: an update. *Contemporary Clinical Trials*.

[12] Egger M., Davey Smith G., Schneider M., Minder C. (1997). Bias in meta-analysis detected by a simple, graphical test. *British Medical Journal*.

[13] Begg C. B., Mazumdar M. (1994). Operating characteristics of a rank correlation test for publication bias. *Biometrics*.

[14] Cavallini G. M., Campi L., Masini C., Pelloni S., Pupino A. (2007). Bimanual microphacoemulsification versus coaxial miniphacoemulsification: prospective study. *Journal of Cataract and Refractive Surgery*.

[15] Elkady B., Pinero D., Alio J. L. (2009). Corneal incision quality: microincision cataract surgery versus microcoaxial phacoemulsification. *Journal of Cataract and Refractive Surgery*.

[16] Can I., Takmaz T., Yildiz Y., Bayhan H. A., Soyugelen G., Bostanci B. (2010). Coaxial, microcoaxial, and biaxial microincision cataract surgery: prospective comparative study. *Journal of Cataract and Refractive Surgery*.

[17] Can I., Bayhan H. A., Celik H., Bostanci Ceran B. (2011). Anterior segment optical coherence tomography evaluation and comparison of main clear corneal incisions in microcoaxial and biaxial cataract surgery. *Journal of Cataract and Refractive Surgery*.

[18] Can I., Bayhan H. A., Celik H., Ceran B. B. (2012). Comparison of corneal aberrations after biaxial microincision and microcoaxial cataract surgeries: a prospective study. *Current Eye Research*.

[19] Alió J. L., Soria F., Abdou A. A., Peña-García P., Fernández-Buenaga R., Javaloy J. (2014). Comparative outcomes of bimanual MICS and 2.2-mm coaxial phacoemulsification assisted by femtosecond technology. *Journal of Refractive Surgery*.

[20] Wilczynski M., Supady E., Loba P., Synder A., Palenga-Pydyn D., Omulecki W. (2009). Comparison of early corneal endothelial cell loss after coaxial phacoemulsification through 1.8 mm microincision and bimanual phacoemulsification through 1.7 mm microincision. *Journal of Cataract and Refractive Surgery*.

[21] Wilczynski M., Supady E., Piotr L., Synder A., Palenga-Pydyn D., Omulecki W. (2009). Comparison of surgically induced astigmatism after coaxial phacoemulsification through 1.8 mm microincision and bimanual phacoemulsification through 1.7 mm microincision. *Journal of Cataract and Refractive Surgery*.

[22] Fine I. H., Hoffman R. S., Packer M. (2004). Optimizing refractive lens exchange with bimanual microincision phacoemulsification. *Journal of Cataract and Refractive Surgery*.

[23] Cavallini G. M. C. L., Torlai G., Forlini M., Fornasari E. (2012). Clear corneal incisions in bimanual microincision cataract surgery: long-term wound-healing architecture. *Journal of Cataract and Refractive Surgery*.

[24] Behrens A., Stark W. J., Pratzer K. A., McDonnell P. J. (2008). Dynamics of small-incision clear cornea wounds after phacoemulsification surgery using optical coherence tomography in the early postoperative period. *Journal of Refractive Surgery*.

[25] Chee S.-P., Bacsal K. (2005). Endophthalmitis after microincision cataract surgery. *Journal of Cataract and Refractive Surgery*.

[26] Alio J. L., Elkady B., Ortiz D. (2010). Corneal optical quality following sub 1.8 mm micro-incision cataract surgery vs. 2.2 mm mini-incision coaxial phacoemulsification. *Middle East African Journal of Ophthalmology*.

[27] Jiang Y., Le Q., Yang J., Lu Y. (2006). Changes in corneal astigmatism and high order aberrations after clear corneal tunnel phacoemulsification guided by corneal topography. *Journal of Refractive Surgery*.

[28] Yao K., Tang X., Ye P. (2006). Corneal astigmatism, high order aberrations, and optical quality after cataract surgery: microincision versus small incision. *Journal of Refractive Surgery*.

[29] Eliwa T. F. E. M., Hamza I. (2015). Effect of biaxial versus coaxial microincision cataract surgery on optical quality of the cornea. *Indian Journal of Ophthalmology*.

[30] Alio J. L. R. P. J., Galal A. (2004). *MICS: MicroIncision Cataract Surgery*.

[31] Alio J. L. F. H. (2010). *Minimizing Incisions and Maximizing Outcomes in Cataract Surgery*.

[32] Cavallini G. M., Verdina T., Forlini M. (2016). Long-term follow-up for bimanual microincision cataract surgery: comparison of results obtained by surgeons in training and experienced surgeons. *Clinical Ophthalmology*.

[33] Cavallini G. M., Volante V., Verdina T. (2015). Results and complications of surgeons-in-training learning bimanual microincision cataract surgery. *Journal of Cataract and Refractive Surgery*.

